# Crystal structures of 4-bromo-2-formyl-1-tosyl-1*H*-pyrrole, (*E*)-4-bromo-2-(2-nitro­vin­yl)-1-tosyl-1*H*-pyrrole and 6-(4-bromo-1-tosyl­pyrrol-2-yl)-4,4-dimethyl-5-nitro­hexan-2-one

**DOI:** 10.1107/S2056989021002280

**Published:** 2021-03-05

**Authors:** Christopher J. Kingsbury, Harry C. Sample, Mathias O. Senge

**Affiliations:** a Chair of Organic Chemistry, School of Chemistry, Trinity Biomedical Science Institute, 152-160 Pearse Street, Trinity College Dublin, The University of Dublin, Dublin 2, Ireland

**Keywords:** crystal structure, pyrrole, chemical inter­mediates, high-resolution

## Abstract

The crystal structures of three inter­mediate compounds in the synthesis of 8-bromo-2,3,4,5-tetra­hydro-1,3,3-tri­methyl­dipyrrin are reported; 4-bromo-2-formyl-1-tosyl-1*H*-pyrrole, C_12_H_10_BrNO_3_S, (*E*)-4-bromo-2-(2-nitro­vin­yl)-1-tosyl-1*H*-pyrrole, C_13_H_11_BrN_2_O_4_S, and 6-(4-bromo-1-tosyl­pyrrol-2-yl)-4,4-dimethyl-5-nitro­hexan-2-one, C_19_H_23_BrN_2_O_5_S. The compounds show multitudinous inter­molecular C—H⋯O inter­actions, with bond distances and angle consistent in the series and within expectations, as well as varied packing types. The merits of collecting data beyond the standard resolution usually reported for small mol­ecules are discussed.

## Abstract 2

Crystal structures of three substituted *N*-tosyl­pyrrole compounds are reported; these compounds show a variety of ‘weak’ inter­molecular inter­actions owing to different substitution patterns and supra­molecular arrangements. The benefits of collecting crystal structure data to extreme resolution (0.5 Å) are discussed.

## Chemical context   

Dipyrrins – 2,2′-dipyrromethenes – are mol­ecular building blocks for multi-pyrrole fluoro­phores such as BODIPYs and porphyrins (*e.g.*, Boyle *et al.*, 1999 ▸) employed as ligands in medicinal and materials chemistry (*e.g*., Hohlfeld *et al.*, 2021 ▸) made through facile condensation reactions, and widely exploited in chemistry. Partially reduced analogues of dipyrrins, containing one pyrrole and one pyrroline unit, are conceptually similar to chlorins – *e.g.* chloro­phylls – where reduction of a macrocycle bond introduces electronic and photophysical changes (Senge *et al.*, 2014 ▸). Synthetic chlorins are produced throught these inter­mediates by stepwise formation of a pyrroline ring (Taniguchi & Lindsey, 2017 ▸), pioneered by Battersby and coworkers (Dutton *et al.*, 1983 ▸) and refined by Lindsey and coworkers (Laha *et al.*, 2006 ▸). The compounds presented here are inter­mediates in the synthesis of derivatives of tetra­hydro­dipyrrin **4**, a versatile precursor that can be formed in high yield from inexpensive reagents.

## Structural commentary   

**Figure 1:**
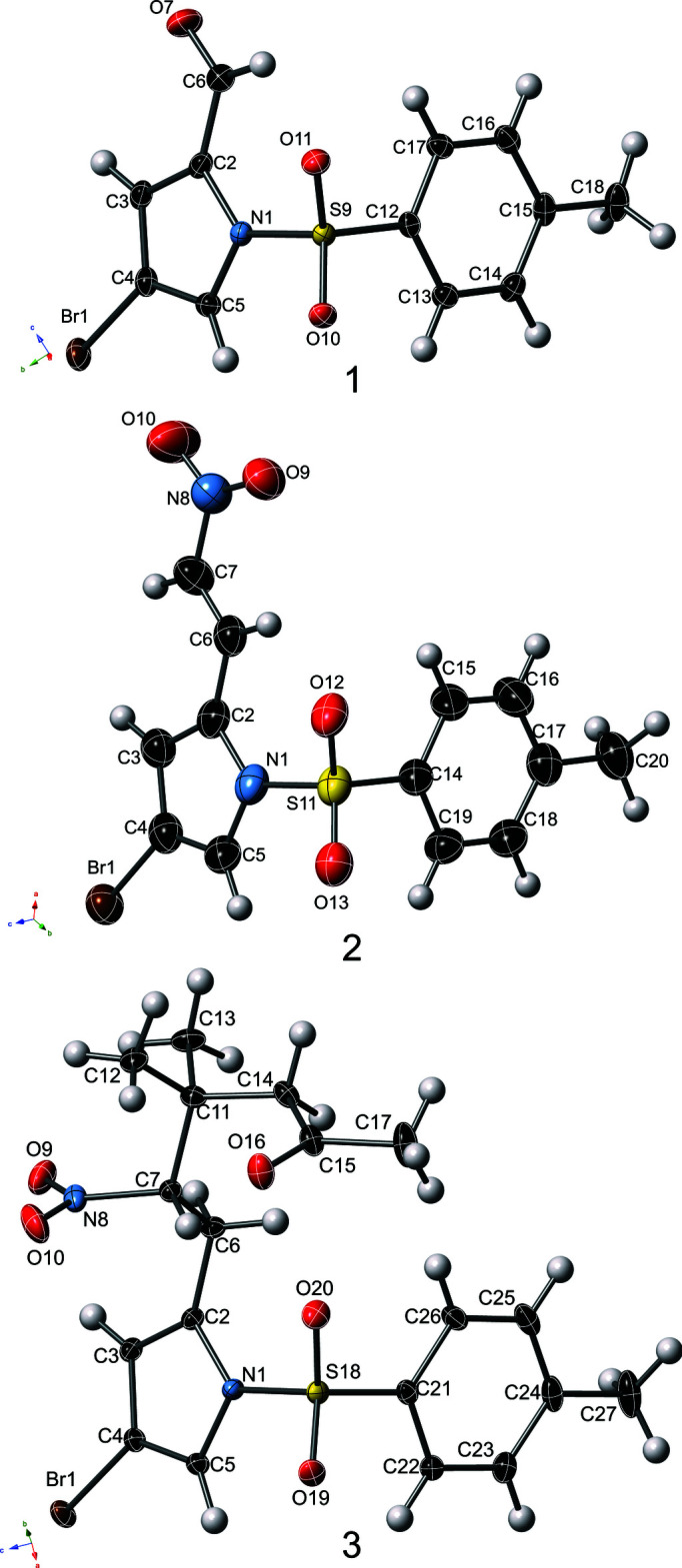
*ORTEP* plots of the mol­ecular units in the crystal structures of compounds **1**, **2** and **3**. Displacement ellipsoids (non-H) are presented at the 50% probability level, with H atoms presented as spheres of fixed radius (0.2 Å).

The crystal structures of **1**, **2**, and **3** (see Scheme and Fig. 1 ▸) each display an isolated mol­ecule with no solvate included, with *Z* = 2 (for **2**) and *Z* = 4 (for **1** and **3**). Each mol­ecular structure shows a 2-substituted-4-bromo-1-tosyl-1*H*-pyrrole, with the 2-substitution as an aldehyde (**1**, *R* = CHO), a 2-nitro­vinyl [**2**, *R* = (*E*)-(CH)_2_NO_2_] and a 3,3-dimethyl-2-nitro­hexan-5-one substituent (**3**). The pyrrole fragment presents approximately consistent inter­nal bond distances throughout this series, as demonstrated in Table 1 ▸. The pyrrole and tosyl groups adopt a consistent conformational structure with N—S and N—C bond torsion angles each at approximately 90°, as discussed in the *Database survey* section.
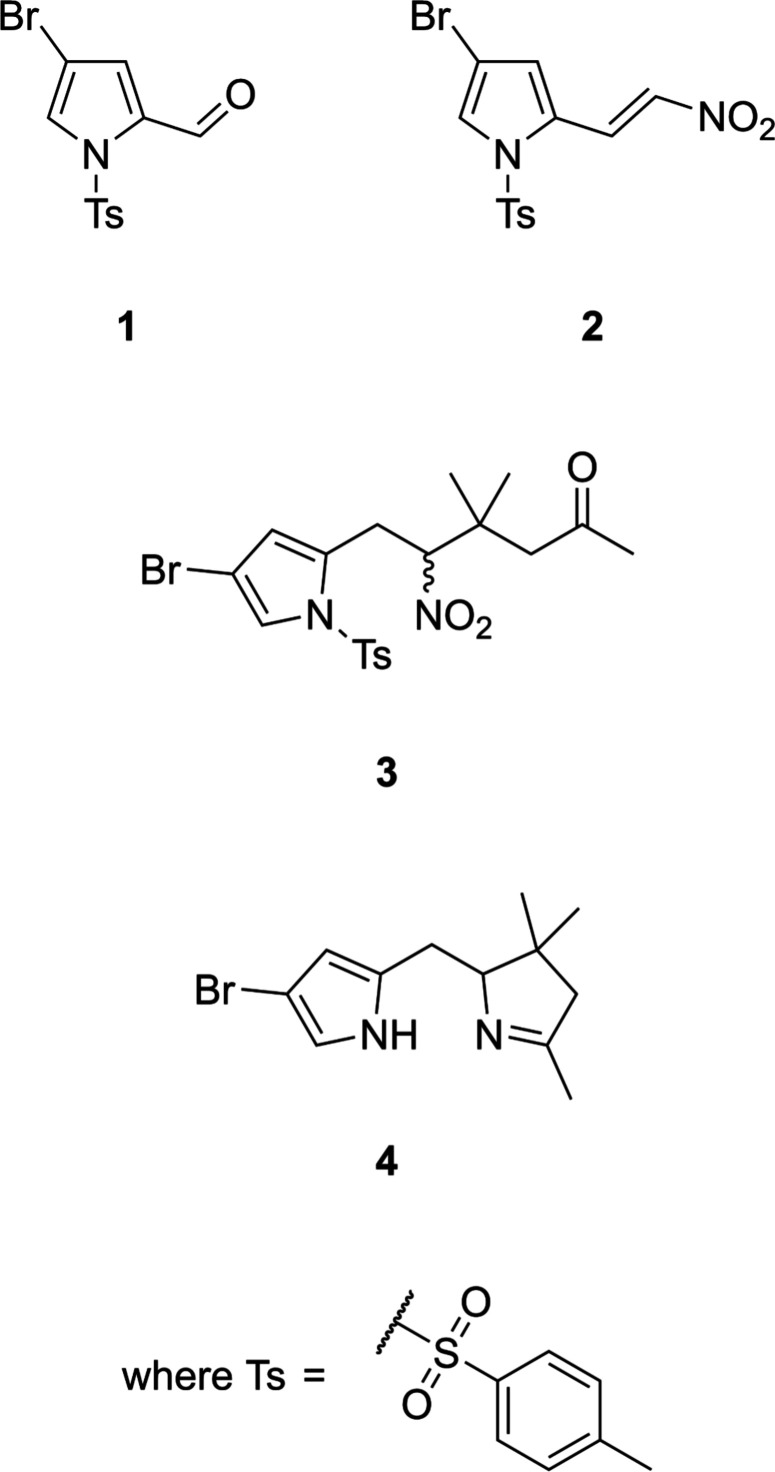


Compound **1** crystallizes in the chiral space group *P*2_1_2_1_2_1_; although this compound exhibits no individual chiral atom centre, the pyrrole and toluene­sulfonyl groups can have many possible orientations, with positive and negative rotation around the N—S bond breaking hypothetical reflection symmetry. The demands of the space-group symmetry of *P*2_1_2_1_2_1_ with *Z*′ = 1 are such that only one of these conformations is found in the unit cell. A Flack parameter of −0.016 (2), although anomalously low, strongly suggests that this individual crystal consists only of this pseudo-atropisomer. No evidence of any barrier to inversion is implied in solution, and enrichment of a preferred orientation in the solid state for this inter­mediate, without similar packing observed for other compounds here, underscores the difficulty in predicting solid-state conformations.

Compound **2** shows comparatively larger displacement ellipsoids than compounds **1** and **3**, but excellent agreement between observations and model, simply without the excessive-resolution data. Compound **3** is the only compound in this series to exhibit a chiral centre – both enanti­omers exist within the unit cell, as this is a conglomerate structure (Viedma *et al.*, 2015 ▸). Both stereoisomers will form identical cyclized (oxidised) products upon conversion to compound **4** or similar species.

## Supra­molecular features   

Each example reported here has a different mode of inter­actions with neighbouring mol­ecules, with no consistent packing in the crystalline solid state. With a lack of heteroatom-bound protons, the solid-state architectures of each of these compounds lack traditional protic structure-directing mortar. Common features are the traditionally overlooked inter­molecular C—H⋯O and C—H⋯Br inter­actions, from the H atoms on the pyrrolyl, vinyl and aryl units to oxygen atoms in the sulfonyl, nitro or ketone moieties. This type of inter­action is assisted by the partial charge separation in these components (Steiner, 2002 ▸).

**Figure 2:**
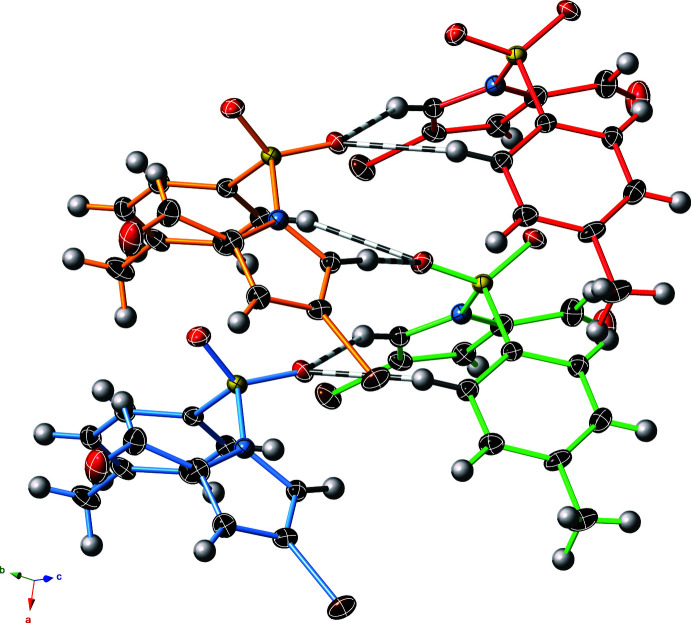
Inter­molecular C—H⋯O inter­actions which control the inter­molecular packing of compound **1**. Displacement ellipsoids are shown at 50% for non-H atoms. Four equivalent mol­ecules – in red, orange, green and blue – are related by a 2_1_ screw coincident with the *a* axis.

Individual mol­ecules of compound **1** stack directly on top of one another down the crystallographic *a-*axis direction, and show a C—H⋯O chelate to mol­ecules in an adjacent stack (Table 2 ▸), related by the 2_1_ screw coincident with the *a* axis. This inter­action is shown in Fig. 2 ▸. Compound **2** shows coplanar inter­molecular inter­actions of the nitro­vinyl­pyrrole unit (Table 3 ▸), in which short contacts can be observed as a C—H⋯O pseudo-chelate (3.36 and 3.30 Å, C⋯O), as well as C—H⋯Br (3.84 Å) inter­actions at the limit of notability. These two inter­actions serve to form ribbon-like arrangements, which propagate coincident with the crystallographic axes [2
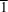
0] vector. Compound **3** demonstrates C—H⋯O (3.28 and 3.29 Å, C⋯O) and C—H⋯Br (3.88 Å) close-contact inter­actions; due to the length, these are likely superficial rather than structure directing.

In each of the compounds reported here, a multitude of unremarkable inter­actions around the van der Waals limit are observed to constrain individual mol­ecules. The presence of C—H⋯O inter­actions would likely be unremarkable if not for the chelate motif – these so-called weak inter­actions can be far stronger with partial charge separation, such as in a sulfonyl, and when occurring at multiple preorganized sites simultaneously (Kingsbury *et al.*, 2019 ▸). Collection of multiple crystal structures along the synthetic pathway of organic compounds is, we believe, good practice to assist data science investigations, and offers potential insight into the electronic structure of inter­mediates (Senge & Smith, 2005 ▸).

## Database survey   

A search of the Cambridge Structural Database (CSD v 2020.3; Groom *et al.*, 2016 ▸) revealed 37 closely related structures with the 2-carbo-4-halo-pyrrole substructure. These structures can be divided into BODIPYs and analogues (13/37), other isolated organic mol­ecules (23/37), including inter­mediates in the total synthesis of (±)-sceptrin, and a lone Cu coordination complex.

A similar compound HULBIA, a bis­(meth­oxy)methyl derivative of **3** has been reported (Krayer *et al.*, 2009 ▸). The presence of a protecting group at the pyrrole N atom is critical in the performance of metal-catalysed reactions; similar 2-substituted-4-halogenated pyrroles have been formed with different N-substitution of *N*-Boc (UJADUF; Merkul *et al.*, 2009 ▸), with an aesthetic seven-membered cycle (PYAZPC; Flippen & Gilardi, 1974 ▸), and a simple methyl group (FONHOG; Zeng *et al.*, 2005 ▸). The non-tosyl­ated iodo-analogue of **1** (HILTOM; Davis *et al.*, 2007 ▸) has been reported previously.

**Figure 3:**
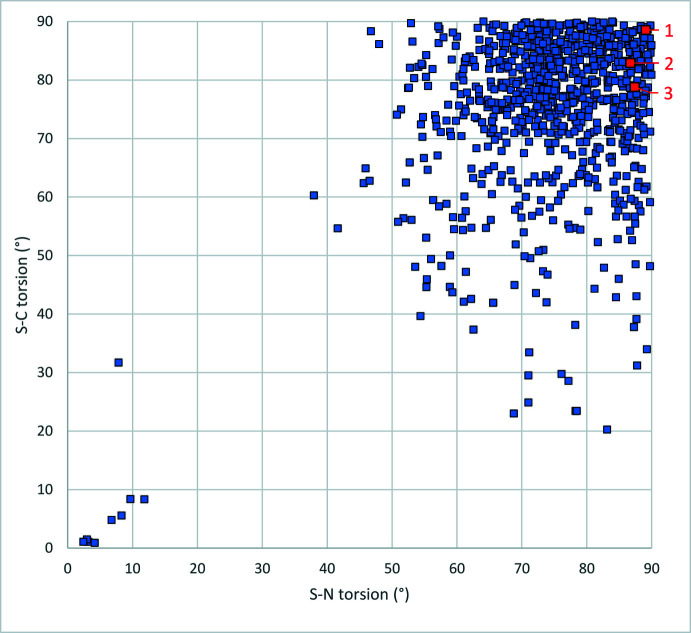
Ramachandran-style plot of torsion angles (°) of central S—C and S—N bonds within *N*-benzene­sulfonyl­pyrrole substructures of crystal structures in the CSD v2020.3 (*n* = 851). Compounds **1**, **2** and **3** are highlighted in red within the main orientation cluster.

A data analysis of a further 851 structures with an *N*-benzene­sulfonyl-pyrrole substructure shows that the component torsional angles (in the range of 0–90°), critcal in determining the solid-state conformation, each tend toward 90°. These values are consistent with our observations of an approximately adjacent-faces-of-a-cube arrangement of these two components. A Ramachandran-style plot illustrating the structural confluence of these two torsion angles is shown in Fig. 3 ▸, with the three compounds presented here highlighted in red.

## Synthesis and crystallization   

The synthesis of these compounds has been previously reported (Krayer *et al.*, 2009 ▸). Crystals of the compounds **1**, **2** and **3** were grown by hot recrystallization from ethyl acetate/hexane mixture (**1**) or iso­propanol (**2**) or slow evaporation of aceto­nitrile (**3**).

## Refinement   

Crystal data, data collection and structure refinement details are summarized in Table 4 ▸.

**Figure 4:**
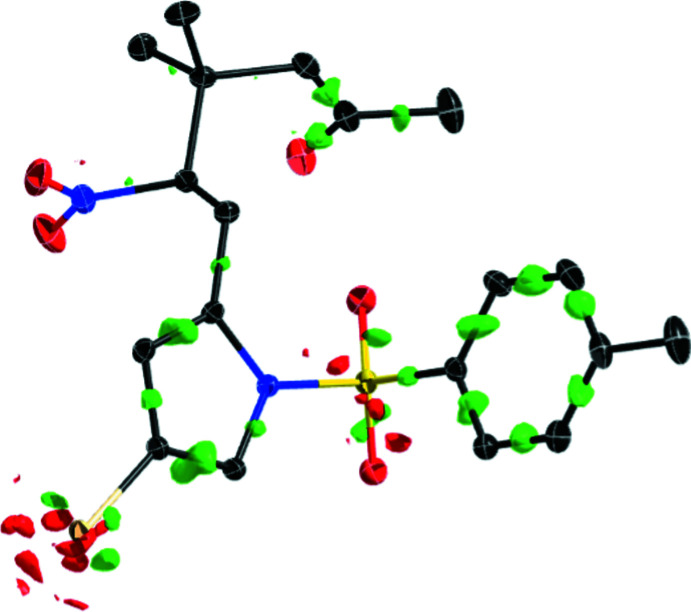
Residual electron density in the high-resolution data structure of **3**; isosurface at 0.4 e^−^ Å^−3^ (+ve in green, -ve in red). H atoms omitted from view. This plot shows residual positive electron density at the centre point of a significant fraction of the C—C bonds.

The collection of high-resolution data (to 0.7 Å for **1** and 0.5 Å for **3**, with Mo *K*α) appears to have an effect on the quality of the structure solution and refinement. Residual electron density at the centre of each bond is apparent, as shown in Fig. 4 ▸; displacement ellipsoids are small. This additional data allows for bond distances to be determined at greater precision, as indicated in Table 1 ▸, and for the time involved in collection of this data to be extended artificially by 3–4 times. While unnecessary, this additional precision merits collection on crystals of sufficient quality when shorter collections are inconvenient. The suppression of presumably non-thermal character of displacement ellipsoids, such as that shown in compound **2**, implies that the true thermal character at cryogenic temperatures is able to be better identified in high-resolution structures, though this could be the coincident effect of additional redundancy.

## Supplementary Material

Crystal structure: contains datablock(s) 1, 2, 3. DOI: 10.1107/S2056989021002280/tx2036sup1.cif

Structure factors: contains datablock(s) 1. DOI: 10.1107/S2056989021002280/tx20361sup2.hkl

Supporting information file. DOI: 10.1107/S2056989021002280/tx20361sup5.cml

Structure factors: contains datablock(s) 2. DOI: 10.1107/S2056989021002280/tx20362sup3.hkl

Supporting information file. DOI: 10.1107/S2056989021002280/tx20362sup6.cml

Structure factors: contains datablock(s) 3. DOI: 10.1107/S2056989021002280/tx20363sup4.hkl

Supporting information file. DOI: 10.1107/S2056989021002280/tx20363sup7.cml

CCDC references: 2065359, 2065358, 2065357

Additional supporting information:  crystallographic information; 3D view; checkCIF report

## Figures and Tables

**Table 1:** Bond distances (Å) in the shared pyrrole fragment of compounds **1**, **2** and **3**

Bond	**1**	**2**	**3**
N1—C2	1.404 (2)	1.399 (4)	1.4054 (7)
C2—C3	1.377 (3)	1.381 (5)	1.3692 (7)
C3—C4	1.410 (3)	1.414 (5)	1.4226 (8)
C4—C5	1.368 (3)	1.361 (5)	1.3613 (8)
C5—N1	1.378 (3)	1.381 (4)	1.3942 (7)
N1—S	1.7002 (16)	1.698 (3)	1.6808 (5)
C4—Br	1.879 (2)	1.881 (3)	1.8727 (5)

**Table 2:** Hydrogen-bond geometry (Å, °) for **1** [Chem scheme1]

*D*—H⋯*A*	*D*—H	H⋯*A*	*D*⋯*A*	*D*—H⋯*A*
C6—H6⋯O11	0.95	2.38	2.994 (3)	122
C5—H5⋯O10^i^	0.95	2.55	3.423 (2)	153
C13—H13⋯O10^i^	0.95	2.56	3.470 (3)	160

Symmetry code: (i) x+{\script{1\over 2}}, -y+{\script{3\over 2}}, -z+1.

**Table 3:** Hydrogen-bond geometry (Å, °) for **2** [Chem scheme1]

*D*—H⋯*A*	*D*—H	H⋯*A*	*D*⋯*A*	*D*—H⋯*A*
C3—H3⋯O10^i^	0.95	2.37	3.297 (5)	166
C7—H7⋯O10^i^	0.95	2.41	3.360 (5)	174
C6—H6⋯O12	0.95	2.29	2.963 (4)	127

Symmetry code: (i) -x, -y+2, -z+1.

**Table 4:** Experimental details

	**1**	**2**	**3**
Crystal data			
Chemical formula	C_12_H_10_BrNO_3_S	C_13_H_11_BrN_2_O_4_S	C_19_H_23_BrN_2_O_5_S
*M*_r_	328.18	371.21	471.36
Crystal system, space group	Orthorhombic, *P*2_1_2_1_2_1_	Triclinic, *P*\overline{1}	Monoclinic, *P*2_1_/*c*
Temperature (K)	100	100	100
*a*, *b*, *c* (Å)	4.8436 (5), 13.9149 (13), 18.5479 (17)	6.8904 (4), 8.3224 (4), 12.8763 (7)	7.7375 (2), 15.9728 (3), 16.7621 (3)
α, β, γ (°)	90, 90, 90	83.423 (3), 80.393 (3), 85.693 (3)	90, 93.055 (1), 90
*V* (Å^3^)	1250.1 (2)	722.06 (7)	2068.68 (8)
*Z*	4	2	4
Radiation type	Mo *K*α	Cu *K*α	Mo *K*α
μ (mm^−1^)	3.45	5.40	2.12
Crystal size (mm)	0.20 × 0.09 × 0.06	0.08 × 0.06 × 0.01	0.61 × 0.56 × 0.55

Data collection			
Diffractometer	Bruker APEXII CCD	Bruker APEXII CCD	Bruker APEXII CCD
Absorption correction	Multi-scan (*SADABS*; Krause *et al.*, 2015 ▸)	Multi-scan (*SADABS*; Krause *et al.*, 2015 ▸)	Multi-scan (*SADABS*; Krause *et al.*, 2015 ▸)
*T*_min_, *T*_max_	0.616, 0.746	0.544, 0.753	0.669, 0.749
No. of measured, independent and observed [*I* > 2σ(*I*)] reflections	23296, 3972, 3714	7190, 2617, 2378	226885, 18433, 15578
*R*_int_	0.028	0.042	0.031
(sin θ/λ)_max_ (Å^−1^)	0.725	0.602	1.021

Refinement			
*R*[*F* ^2^ > 2σ(*F* ^2^)], *wR*(*F* ^2^), *S*	0.021, 0.045, 1.04	0.049, 0.142, 1.05	0.027, 0.076, 1.11
No. of reflections	3972	2617	18433
No. of parameters	164	191	257
H-atom treatment	H-atom parameters constrained	H-atom parameters constrained	H-atom parameters constrained
Δρ_max_, Δρ_min_ (e Å^−3^)	0.34, −0.38	0.80, −0.63	0.69, −0.72
Absolute structure	Flack *x* determined using 1452 quotients [(*I* ^+^)−(*I* ^−^)]/[(*I* ^+^)+(*I* ^−^)] (Parsons *et al.*, 2013 ▸)	–	–
Absolute structure parameter	−0.016 (2)	–	–

Computer programs: *APEX3* and *SAINT* (Bruker, 2015 ▸), *SHELXT2018/2* (Sheldrick, 2015*a* ▸), *SHELXL2018/3* (Sheldrick, 2015*b* ▸), *shelXle* (Hübschle *et al.*, 2011 ▸) and *publCIF* (Westrip, 2010 ▸).

## supplementary crystallographic information

### 4-Bromo-1-[(4-methylbenzene)sulfonyl]pyrrole-2-carbaldehyde (1) Crystal data

**Table:**

C_12_H_10_BrNO_3_S	*D*_x_ = 1.744 Mg m^−^^3^
*M**_r_* = 328.18	Mo *K*α radiation, λ = 0.71073 Å
Orthorhombic, *P*2_1_2_1_2_1_	Cell parameters from 9913 reflections
*a* = 4.8436 (5) Å	θ = 2.9–31.4°
*b* = 13.9149 (13) Å	µ = 3.45 mm^−^^1^
*c* = 18.5479 (17) Å	*T* = 100 K
*V* = 1250.1 (2) Å^3^	Rod, colourless
*Z* = 4	0.20 × 0.09 × 0.06 mm
*F*(000) = 656	

### 4-Bromo-1-[(4-methylbenzene)sulfonyl]pyrrole-2-carbaldehyde (1) Data collection

**Table:**

Bruker APEXII CCD diffractometer	3714 reflections with *I* > 2σ(*I*)
φ and ω scans	*R*_int_ = 0.028
Absorption correction: multi-scan (SADABS; Krause *et al.*, 2015)	θ_max_ = 31.0°, θ_min_ = 1.8°
*T*_min_ = 0.616, *T*_max_ = 0.746	*h* = −7→6
23296 measured reflections	*k* = −20→19
3972 independent reflections	*l* = −20→26

### 4-Bromo-1-[(4-methylbenzene)sulfonyl]pyrrole-2-carbaldehyde (1) Refinement

**Table:**

Refinement on *F*^2^	Primary atom site location: structure-invariant direct methods
Least-squares matrix: full	Hydrogen site location: inferred from neighbouring sites
*R*[*F*^2^ > 2σ(*F*^2^)] = 0.021	H-atom parameters constrained
*wR*(*F*^2^) = 0.045	*w* = 1/[σ^2^(*F*_o_^2^) + (0.0201*P*)^2^ + 0.4002*P*] where *P* = (*F*_o_^2^ + 2*F*_c_^2^)/3
*S* = 1.04	(Δ/σ)_max_ = 0.001
3972 reflections	Δρ_max_ = 0.34 e Å^−^^3^
164 parameters	Δρ_min_ = −0.38 e Å^−^^3^
0 restraints	

### 4-Bromo-1-[(4-methylbenzene)sulfonyl]pyrrole-2-carbaldehyde (1) Special details

**Table:**

Geometry. All esds (except the esd in the dihedral angle between two l.s. planes) are estimated using the full covariance matrix. The cell esds are taken into account individually in the estimation of esds in distances, angles and torsion angles; correlations between esds in cell parameters are only used when they are defined by crystal symmetry. An approximate (isotropic) treatment of cell esds is used for estimating esds involving l.s. planes.

### 4-Bromo-1-[(4-methylbenzene)sulfonyl]pyrrole-2-carbaldehyde (1) Fractional atomic coordinates and isotropic or equivalent isotropic displacement parameters (Å^2^)

**Table:**

	*x*	*y*	*z*	*U*_iso_*/*U*_eq_	
Br1	1.03533 (5)	0.95495 (2)	0.67018 (2)	0.02243 (6)	
N1	0.5067 (3)	0.73027 (11)	0.66274 (9)	0.0138 (3)	
C2	0.5792 (4)	0.72511 (15)	0.73590 (11)	0.0167 (4)	
C3	0.7636 (4)	0.79838 (15)	0.74892 (11)	0.0181 (4)	
H3	0.847177	0.813140	0.793908	0.022*	
C4	0.8059 (4)	0.84751 (15)	0.68338 (10)	0.0163 (4)	
C5	0.6504 (4)	0.80452 (15)	0.63092 (11)	0.0153 (4)	
H5	0.642846	0.822553	0.581559	0.018*	
C6	0.4882 (5)	0.65241 (16)	0.78783 (11)	0.0226 (4)	
H6	0.357430	0.605380	0.773252	0.027*	
O7	0.5766 (4)	0.65095 (14)	0.84935 (8)	0.0334 (4)	
S9	0.30100 (10)	0.65428 (4)	0.61513 (3)	0.01313 (9)	
O10	0.2261 (3)	0.70813 (11)	0.55234 (7)	0.0167 (3)	
O11	0.0978 (3)	0.62059 (10)	0.66500 (8)	0.0175 (3)	
C12	0.5188 (4)	0.55963 (13)	0.59054 (10)	0.0135 (3)	
C13	0.6698 (4)	0.56650 (15)	0.52637 (11)	0.0170 (4)	
H13	0.653329	0.621364	0.496191	0.020*	
C14	0.8442 (5)	0.49124 (16)	0.50790 (12)	0.0187 (4)	
H14	0.947090	0.494838	0.464384	0.022*	
C15	0.8716 (4)	0.41035 (16)	0.55192 (12)	0.0182 (4)	
C16	0.7162 (5)	0.40561 (16)	0.61547 (12)	0.0189 (4)	
H16	0.731639	0.350655	0.645586	0.023*	
C17	0.5402 (5)	0.47959 (14)	0.63526 (11)	0.0168 (4)	
H17	0.436014	0.475763	0.678556	0.020*	
C18	1.0659 (5)	0.33074 (16)	0.53061 (12)	0.0236 (5)	
H18A	1.020615	0.308589	0.481867	0.035*	
H18B	1.047418	0.277176	0.564571	0.035*	
H18C	1.256213	0.354656	0.531514	0.035*	

### 4-Bromo-1-[(4-methylbenzene)sulfonyl]pyrrole-2-carbaldehyde (1) Atomic displacement parameters (Å^2^)

**Table:**

	*U*^11^	*U*^22^	*U*^33^	*U*^12^	*U*^13^	*U*^23^
Br1	0.02171 (10)	0.01959 (10)	0.02598 (10)	−0.00630 (9)	0.00443 (8)	−0.00624 (9)
N1	0.0142 (8)	0.0138 (7)	0.0134 (7)	−0.0004 (6)	−0.0005 (7)	−0.0014 (6)
C2	0.0185 (10)	0.0186 (10)	0.0128 (8)	0.0029 (8)	−0.0004 (7)	−0.0026 (7)
C3	0.0192 (10)	0.0193 (10)	0.0159 (9)	0.0014 (8)	−0.0004 (8)	−0.0050 (8)
C4	0.0146 (9)	0.0150 (9)	0.0194 (10)	−0.0004 (8)	0.0021 (7)	−0.0045 (7)
C5	0.0149 (9)	0.0155 (10)	0.0155 (9)	0.0010 (7)	0.0012 (7)	0.0009 (7)
C6	0.0289 (12)	0.0209 (10)	0.0182 (9)	−0.0009 (10)	−0.0002 (9)	−0.0004 (8)
O7	0.0507 (12)	0.0331 (9)	0.0166 (7)	−0.0048 (9)	−0.0060 (7)	0.0037 (7)
S9	0.0118 (2)	0.0141 (2)	0.0135 (2)	0.00034 (18)	−0.00095 (17)	−0.00007 (17)
O10	0.0166 (7)	0.0179 (7)	0.0154 (7)	0.0011 (6)	−0.0039 (5)	0.0015 (6)
O11	0.0135 (7)	0.0210 (7)	0.0181 (7)	−0.0011 (5)	0.0031 (6)	0.0006 (6)
C12	0.0123 (8)	0.0125 (9)	0.0156 (8)	0.0001 (7)	−0.0010 (7)	−0.0015 (6)
C13	0.0172 (10)	0.0170 (10)	0.0168 (9)	0.0011 (8)	−0.0007 (7)	0.0005 (7)
C14	0.0177 (10)	0.0218 (10)	0.0165 (9)	0.0008 (8)	0.0005 (8)	−0.0040 (8)
C15	0.0148 (9)	0.0182 (10)	0.0215 (10)	0.0007 (8)	−0.0070 (7)	−0.0068 (8)
C16	0.0191 (10)	0.0168 (10)	0.0208 (10)	0.0015 (8)	−0.0056 (8)	0.0012 (8)
C17	0.0166 (9)	0.0167 (9)	0.0170 (9)	−0.0011 (8)	−0.0015 (8)	0.0014 (7)
C18	0.0202 (11)	0.0226 (11)	0.0280 (11)	0.0045 (9)	−0.0076 (9)	−0.0094 (9)

### 4-Bromo-1-[(4-methylbenzene)sulfonyl]pyrrole-2-carbaldehyde (1) Geometric parameters (Å, º)

**Table:**

Br1—C4	1.879 (2)	C12—C17	1.393 (3)
N1—C5	1.378 (3)	C12—C13	1.400 (3)
N1—C2	1.404 (2)	C13—C14	1.388 (3)
N1—S9	1.7002 (16)	C13—H13	0.9500
C2—C3	1.377 (3)	C14—C15	1.397 (3)
C2—C6	1.465 (3)	C14—H14	0.9500
C3—C4	1.410 (3)	C15—C16	1.400 (3)
C3—H3	0.9500	C15—C18	1.506 (3)
C4—C5	1.368 (3)	C16—C17	1.386 (3)
C5—H5	0.9500	C16—H16	0.9500
C6—O7	1.219 (3)	C17—H17	0.9500
C6—H6	0.9500	C18—H18A	0.9800
S9—O11	1.4296 (15)	C18—H18B	0.9800
S9—O10	1.4316 (15)	C18—H18C	0.9800
S9—C12	1.7481 (19)		
			
C5—N1—C2	109.04 (16)	C17—C12—C13	121.47 (18)
C5—N1—S9	122.66 (14)	C17—C12—S9	119.47 (15)
C2—N1—S9	128.08 (14)	C13—C12—S9	119.05 (15)
C3—C2—N1	107.09 (18)	C14—C13—C12	118.42 (19)
C3—C2—C6	126.25 (19)	C14—C13—H13	120.8
N1—C2—C6	126.58 (19)	C12—C13—H13	120.8
C2—C3—C4	107.59 (18)	C13—C14—C15	121.4 (2)
C2—C3—H3	126.2	C13—C14—H14	119.3
C4—C3—H3	126.2	C15—C14—H14	119.3
C5—C4—C3	108.74 (19)	C16—C15—C14	118.6 (2)
C5—C4—Br1	125.50 (16)	C16—C15—C18	121.5 (2)
C3—C4—Br1	125.75 (15)	C14—C15—C18	119.9 (2)
C4—C5—N1	107.52 (17)	C17—C16—C15	121.2 (2)
C4—C5—H5	126.2	C17—C16—H16	119.4
N1—C5—H5	126.2	C15—C16—H16	119.4
O7—C6—C2	121.4 (2)	C16—C17—C12	118.81 (19)
O7—C6—H6	119.3	C16—C17—H17	120.6
C2—C6—H6	119.3	C12—C17—H17	120.6
O11—S9—O10	121.57 (9)	C15—C18—H18A	109.5
O11—S9—N1	105.75 (9)	C15—C18—H18B	109.5
O10—S9—N1	104.20 (9)	H18A—C18—H18B	109.5
O11—S9—C12	109.72 (9)	C15—C18—H18C	109.5
O10—S9—C12	109.57 (9)	H18A—C18—H18C	109.5
N1—S9—C12	104.50 (9)	H18B—C18—H18C	109.5
			
C5—N1—C2—C3	1.4 (2)	C5—N1—S9—C12	90.92 (17)
S9—N1—C2—C3	176.08 (15)	C2—N1—S9—C12	−83.10 (19)
C5—N1—C2—C6	−175.5 (2)	O11—S9—C12—C17	−21.53 (19)
S9—N1—C2—C6	−0.8 (3)	O10—S9—C12—C17	−157.39 (16)
N1—C2—C3—C4	−0.7 (2)	N1—S9—C12—C17	91.45 (17)
C6—C2—C3—C4	176.3 (2)	O11—S9—C12—C13	158.96 (16)
C2—C3—C4—C5	−0.3 (2)	O10—S9—C12—C13	23.10 (19)
C2—C3—C4—Br1	179.24 (16)	N1—S9—C12—C13	−88.05 (17)
C3—C4—C5—N1	1.2 (2)	C17—C12—C13—C14	−0.2 (3)
Br1—C4—C5—N1	−178.37 (14)	S9—C12—C13—C14	179.33 (16)
C2—N1—C5—C4	−1.6 (2)	C12—C13—C14—C15	−0.3 (3)
S9—N1—C5—C4	−176.62 (14)	C13—C14—C15—C16	0.7 (3)
C3—C2—C6—O7	−0.9 (4)	C13—C14—C15—C18	−179.1 (2)
N1—C2—C6—O7	175.5 (2)	C14—C15—C16—C17	−0.6 (3)
C5—N1—S9—O11	−153.29 (16)	C18—C15—C16—C17	179.2 (2)
C2—N1—S9—O11	32.69 (19)	C15—C16—C17—C12	0.2 (3)
C5—N1—S9—O10	−24.06 (18)	C13—C12—C17—C16	0.2 (3)
C2—N1—S9—O10	161.93 (17)	S9—C12—C17—C16	−179.26 (16)

### 4-Bromo-1-[(4-methylbenzene)sulfonyl]pyrrole-2-carbaldehyde (1) Hydrogen-bond geometry (Å, º)

**Table:**

*D*—H···*A*	*D*—H	H···*A*	*D*···*A*	*D*—H···*A*
C6—H6···O11	0.95	2.38	2.994 (3)	122
C5—H5···O10^i^	0.95	2.55	3.423 (2)	153
C13—H13···O10^i^	0.95	2.56	3.470 (3)	160

Symmetry code: (i) *x*+1/2, −*y*+3/2, −*z*+1.

### (E)-4-bromo-2-(2-nitrovinyl)-1-tosyl-1H-pyrrole (2) Crystal data

**Table:**

C_13_H_11_BrN_2_O_4_S	*Z* = 2
*M**_r_* = 371.21	*F*(000) = 372
Triclinic, *P*1	*D*_x_ = 1.707 Mg m^−^^3^
*a* = 6.8904 (4) Å	Cu *K*α radiation, λ = 1.54178 Å
*b* = 8.3224 (4) Å	Cell parameters from 4853 reflections
*c* = 12.8763 (7) Å	θ = 3.5–68.2°
α = 83.423 (3)°	µ = 5.40 mm^−^^1^
β = 80.393 (3)°	*T* = 100 K
γ = 85.693 (3)°	Plate, colourless
*V* = 722.06 (7) Å^3^	0.08 × 0.06 × 0.01 mm

### (E)-4-bromo-2-(2-nitrovinyl)-1-tosyl-1H-pyrrole (2) Data collection

**Table:**

Bruker APEXII CCD diffractometer	2378 reflections with *I* > 2σ(*I*)
φ and ω scans	*R*_int_ = 0.042
Absorption correction: multi-scan (SADABS; Krause *et al.*, 2015)	θ_max_ = 68.2°, θ_min_ = 3.5°
*T*_min_ = 0.544, *T*_max_ = 0.753	*h* = −6→8
7190 measured reflections	*k* = −9→9
2617 independent reflections	*l* = −15→15

### (E)-4-bromo-2-(2-nitrovinyl)-1-tosyl-1H-pyrrole (2) Refinement

**Table:**

Refinement on *F*^2^	Primary atom site location: structure-invariant direct methods
Least-squares matrix: full	Hydrogen site location: inferred from neighbouring sites
*R*[*F*^2^ > 2σ(*F*^2^)] = 0.049	H-atom parameters constrained
*wR*(*F*^2^) = 0.142	*w* = 1/[σ^2^(*F*_o_^2^) + (0.1118*P*)^2^] where *P* = (*F*_o_^2^ + 2*F*_c_^2^)/3
*S* = 1.05	(Δ/σ)_max_ < 0.001
2617 reflections	Δρ_max_ = 0.80 e Å^−^^3^
191 parameters	Δρ_min_ = −0.63 e Å^−^^3^
0 restraints	

### (E)-4-bromo-2-(2-nitrovinyl)-1-tosyl-1H-pyrrole (2) Special details

**Table:**

Geometry. All esds (except the esd in the dihedral angle between two l.s. planes) are estimated using the full covariance matrix. The cell esds are taken into account individually in the estimation of esds in distances, angles and torsion angles; correlations between esds in cell parameters are only used when they are defined by crystal symmetry. An approximate (isotropic) treatment of cell esds is used for estimating esds involving l.s. planes.

### (E)-4-bromo-2-(2-nitrovinyl)-1-tosyl-1H-pyrrole (2) Fractional atomic coordinates and isotropic or equivalent isotropic displacement parameters (Å^2^)

**Table:**

	*x*	*y*	*z*	*U*_iso_*/*U*_eq_	
Br1	0.78111 (5)	0.59378 (4)	0.35381 (3)	0.0425 (2)	
N1	0.6292 (5)	0.7256 (3)	0.6502 (2)	0.0384 (6)	
C2	0.4649 (5)	0.7946 (4)	0.6072 (3)	0.0358 (7)	
C3	0.4914 (5)	0.7634 (4)	0.5026 (3)	0.0392 (7)	
H3	0.405169	0.797263	0.452898	0.047*	
C4	0.6723 (5)	0.6710 (4)	0.4840 (3)	0.0388 (7)	
C5	0.7544 (6)	0.6478 (4)	0.5740 (3)	0.0397 (7)	
H5	0.875264	0.589067	0.582710	0.048*	
C6	0.3020 (5)	0.8827 (4)	0.6651 (3)	0.0381 (7)	
H6	0.306219	0.898197	0.736623	0.046*	
C7	0.1474 (6)	0.9425 (5)	0.6225 (3)	0.0437 (8)	
H7	0.141618	0.927123	0.551082	0.052*	
N8	−0.0128 (5)	1.0308 (4)	0.6821 (3)	0.0447 (7)	
O9	−0.0096 (4)	1.0452 (4)	0.7764 (2)	0.0499 (6)	
O10	−0.1458 (5)	1.0858 (5)	0.6356 (3)	0.0706 (10)	
S11	0.66335 (12)	0.69881 (8)	0.77875 (6)	0.0366 (2)	
O12	0.5680 (4)	0.8370 (3)	0.8260 (2)	0.0435 (6)	
O13	0.8708 (4)	0.6635 (3)	0.7737 (2)	0.0429 (6)	
C14	0.5375 (5)	0.5261 (4)	0.8327 (2)	0.0338 (6)	
C15	0.3465 (6)	0.5434 (4)	0.8867 (3)	0.0469 (8)	
H15	0.283748	0.647790	0.895024	0.056*	
C16	0.2498 (6)	0.4049 (5)	0.9280 (3)	0.0503 (9)	
H16	0.118816	0.415207	0.964877	0.060*	
C17	0.3389 (6)	0.2510 (4)	0.9171 (3)	0.0408 (7)	
C18	0.5283 (5)	0.2385 (4)	0.8610 (3)	0.0395 (7)	
H18	0.589695	0.134135	0.851045	0.047*	
C19	0.6306 (5)	0.3744 (4)	0.8189 (3)	0.0386 (7)	
H19	0.761277	0.364040	0.781580	0.046*	
C20	0.2280 (6)	0.1045 (4)	0.9648 (3)	0.0480 (9)	
H20A	0.123086	0.091657	0.924079	0.072*	
H20B	0.169992	0.118266	1.038312	0.072*	
H20C	0.318724	0.007925	0.963164	0.072*	

### (E)-4-bromo-2-(2-nitrovinyl)-1-tosyl-1H-pyrrole (2) Atomic displacement parameters (Å^2^)

**Table:**

	*U*^11^	*U*^22^	*U*^33^	*U*^12^	*U*^13^	*U*^23^
Br1	0.0488 (3)	0.0434 (3)	0.0347 (3)	−0.00224 (17)	−0.00398 (17)	−0.00571 (16)
N1	0.0524 (17)	0.0278 (13)	0.0348 (14)	−0.0006 (11)	−0.0084 (12)	−0.0016 (10)
C2	0.0443 (18)	0.0255 (14)	0.0371 (17)	−0.0049 (12)	−0.0072 (14)	0.0015 (12)
C3	0.0442 (18)	0.0341 (16)	0.0370 (17)	−0.0033 (13)	−0.0016 (14)	−0.0004 (12)
C4	0.0500 (19)	0.0289 (15)	0.0360 (16)	−0.0051 (13)	−0.0024 (14)	−0.0020 (12)
C5	0.0501 (19)	0.0293 (15)	0.0381 (17)	0.0016 (12)	−0.0031 (14)	−0.0046 (12)
C6	0.050 (2)	0.0284 (14)	0.0339 (16)	−0.0054 (13)	−0.0027 (14)	−0.0009 (12)
C7	0.0436 (19)	0.0496 (19)	0.0376 (18)	−0.0050 (15)	−0.0016 (14)	−0.0091 (14)
N8	0.0443 (17)	0.0466 (16)	0.0444 (18)	−0.0048 (13)	−0.0077 (14)	−0.0066 (13)
O9	0.0517 (15)	0.0545 (16)	0.0429 (15)	−0.0010 (12)	−0.0034 (12)	−0.0102 (11)
O10	0.0581 (19)	0.099 (3)	0.057 (2)	0.0186 (18)	−0.0181 (15)	−0.0192 (18)
S11	0.0507 (5)	0.0251 (4)	0.0345 (4)	−0.0034 (3)	−0.0070 (3)	−0.0042 (3)
O12	0.0654 (16)	0.0265 (11)	0.0396 (13)	−0.0012 (10)	−0.0100 (11)	−0.0060 (9)
O13	0.0526 (14)	0.0363 (12)	0.0413 (13)	−0.0081 (10)	−0.0100 (11)	−0.0034 (9)
C14	0.0437 (17)	0.0263 (14)	0.0310 (15)	−0.0010 (12)	−0.0031 (12)	−0.0058 (11)
C15	0.058 (2)	0.0318 (16)	0.0441 (19)	0.0063 (14)	0.0081 (16)	−0.0039 (13)
C16	0.048 (2)	0.0415 (19)	0.054 (2)	0.0007 (15)	0.0134 (17)	−0.0068 (15)
C17	0.053 (2)	0.0368 (17)	0.0328 (16)	−0.0071 (14)	−0.0052 (14)	−0.0055 (12)
C18	0.0484 (19)	0.0274 (15)	0.0425 (18)	−0.0015 (12)	−0.0042 (14)	−0.0074 (13)
C19	0.0405 (17)	0.0292 (15)	0.0460 (18)	0.0002 (12)	−0.0062 (14)	−0.0062 (13)
C20	0.061 (2)	0.0407 (18)	0.0410 (19)	−0.0146 (16)	−0.0006 (16)	−0.0045 (14)

### (E)-4-bromo-2-(2-nitrovinyl)-1-tosyl-1H-pyrrole (2) Geometric parameters (Å, º)

**Table:**

Br1—C4	1.881 (3)	S11—O13	1.430 (3)
N1—C5	1.381 (4)	S11—C14	1.748 (3)
N1—C2	1.399 (4)	C14—C15	1.387 (5)
N1—S11	1.698 (3)	C14—C19	1.389 (4)
C2—C3	1.381 (5)	C15—C16	1.383 (6)
C2—C6	1.441 (5)	C15—H15	0.9500
C3—C4	1.414 (5)	C16—C17	1.390 (5)
C3—H3	0.9500	C16—H16	0.9500
C4—C5	1.361 (5)	C17—C18	1.385 (5)
C5—H5	0.9500	C17—C20	1.503 (5)
C6—C7	1.318 (6)	C18—C19	1.386 (5)
C6—H6	0.9500	C18—H18	0.9500
C7—N8	1.439 (5)	C19—H19	0.9500
C7—H7	0.9500	C20—H20A	0.9800
N8—O10	1.210 (5)	C20—H20B	0.9800
N8—O9	1.237 (4)	C20—H20C	0.9800
S11—O12	1.428 (2)		
			
C5—N1—C2	109.1 (3)	O12—S11—C14	109.60 (15)
C5—N1—S11	120.8 (2)	O13—S11—C14	109.53 (15)
C2—N1—S11	129.0 (2)	N1—S11—C14	104.67 (14)
C3—C2—N1	107.6 (3)	C15—C14—C19	121.5 (3)
C3—C2—C6	128.1 (3)	C15—C14—S11	119.5 (2)
N1—C2—C6	124.2 (3)	C19—C14—S11	119.0 (3)
C2—C3—C4	106.5 (3)	C16—C15—C14	118.3 (3)
C2—C3—H3	126.8	C16—C15—H15	120.8
C4—C3—H3	126.8	C14—C15—H15	120.8
C5—C4—C3	109.6 (3)	C15—C16—C17	121.9 (4)
C5—C4—Br1	125.7 (3)	C15—C16—H16	119.1
C3—C4—Br1	124.7 (3)	C17—C16—H16	119.1
C4—C5—N1	107.2 (3)	C18—C17—C16	118.1 (3)
C4—C5—H5	126.4	C18—C17—C20	122.2 (3)
N1—C5—H5	126.4	C16—C17—C20	119.7 (3)
C7—C6—C2	122.4 (4)	C17—C18—C19	121.7 (3)
C7—C6—H6	118.8	C17—C18—H18	119.1
C2—C6—H6	118.8	C19—C18—H18	119.1
C6—C7—N8	121.2 (4)	C18—C19—C14	118.4 (3)
C6—C7—H7	119.4	C18—C19—H19	120.8
N8—C7—H7	119.4	C14—C19—H19	120.8
O10—N8—O9	123.7 (3)	C17—C20—H20A	109.5
O10—N8—C7	116.7 (3)	C17—C20—H20B	109.5
O9—N8—C7	119.6 (3)	H20A—C20—H20B	109.5
O12—S11—O13	121.14 (15)	C17—C20—H20C	109.5
O12—S11—N1	106.10 (14)	H20A—C20—H20C	109.5
O13—S11—N1	104.39 (15)	H20B—C20—H20C	109.5
			
C5—N1—C2—C3	−1.8 (3)	C2—N1—S11—O13	−165.3 (3)
S11—N1—C2—C3	−169.3 (2)	C5—N1—S11—C14	−86.7 (3)
C5—N1—C2—C6	178.8 (3)	C2—N1—S11—C14	79.6 (3)
S11—N1—C2—C6	11.2 (5)	O12—S11—C14—C15	17.3 (3)
N1—C2—C3—C4	1.4 (3)	O13—S11—C14—C15	152.4 (3)
C6—C2—C3—C4	−179.2 (3)	N1—S11—C14—C15	−96.2 (3)
C2—C3—C4—C5	−0.5 (4)	O12—S11—C14—C19	−163.7 (3)
C2—C3—C4—Br1	179.7 (2)	O13—S11—C14—C19	−28.6 (3)
C3—C4—C5—N1	−0.5 (4)	N1—S11—C14—C19	82.9 (3)
Br1—C4—C5—N1	179.2 (2)	C19—C14—C15—C16	0.6 (6)
C2—N1—C5—C4	1.4 (4)	S11—C14—C15—C16	179.6 (3)
S11—N1—C5—C4	170.2 (2)	C14—C15—C16—C17	0.3 (7)
C3—C2—C6—C7	2.4 (6)	C15—C16—C17—C18	−1.5 (6)
N1—C2—C6—C7	−178.3 (3)	C15—C16—C17—C20	179.2 (4)
C2—C6—C7—N8	−179.7 (3)	C16—C17—C18—C19	1.9 (6)
C6—C7—N8—O10	177.7 (4)	C20—C17—C18—C19	−178.8 (3)
C6—C7—N8—O9	−3.0 (6)	C17—C18—C19—C14	−1.0 (5)
C5—N1—S11—O12	157.4 (3)	C15—C14—C19—C18	−0.2 (5)
C2—N1—S11—O12	−36.3 (3)	S11—C14—C19—C18	−179.2 (3)
C5—N1—S11—O13	28.4 (3)		

### (E)-4-bromo-2-(2-nitrovinyl)-1-tosyl-1H-pyrrole (2) Hydrogen-bond geometry (Å, º)

**Table:**

*D*—H···*A*	*D*—H	H···*A*	*D*···*A*	*D*—H···*A*
C3—H3···O10^i^	0.95	2.37	3.297 (5)	166
C7—H7···O10^i^	0.95	2.41	3.360 (5)	174
C6—H6···O12	0.95	2.29	2.963 (4)	127

Symmetry code: (i) −*x*, −*y*+2, −*z*+1.

### 6-(4-bromo-1-tosylpyrrol-2-yl)-4,4-dimethyl-5-nitrohexan-2-one (3) Crystal data

**Table:**

C_19_H_23_BrN_2_O_5_S	*F*(000) = 968
*M**_r_* = 471.36	*D*_x_ = 1.513 Mg m^−^^3^
Monoclinic, *P*2_1_/*c*	Mo *K*α radiation, λ = 0.71073 Å
*a* = 7.7375 (2) Å	Cell parameters from 9692 reflections
*b* = 15.9728 (3) Å	θ = 2.8–46.1°
*c* = 16.7621 (3) Å	µ = 2.12 mm^−^^1^
β = 93.055 (1)°	*T* = 100 K
*V* = 2068.68 (8) Å^3^	Block, colorless
*Z* = 4	0.61 × 0.56 × 0.55 mm

### 6-(4-bromo-1-tosylpyrrol-2-yl)-4,4-dimethyl-5-nitrohexan-2-one (3) Data collection

**Table:**

Bruker APEXII CCD diffractometer	15578 reflections with *I* > 2σ(*I*)
φ and ω scans	*R*_int_ = 0.031
Absorption correction: multi-scan (SADABS; Krause *et al.*, 2015)	θ_max_ = 46.5°, θ_min_ = 1.8°
*T*_min_ = 0.669, *T*_max_ = 0.749	*h* = −15→15
226885 measured reflections	*k* = −32→32
18433 independent reflections	*l* = −34→34

### 6-(4-bromo-1-tosylpyrrol-2-yl)-4,4-dimethyl-5-nitrohexan-2-one (3) Refinement

**Table:**

Refinement on *F*^2^	Primary atom site location: structure-invariant direct methods
Least-squares matrix: full	Hydrogen site location: inferred from neighbouring sites
*R*[*F*^2^ > 2σ(*F*^2^)] = 0.027	H-atom parameters constrained
*wR*(*F*^2^) = 0.076	*w* = 1/[σ^2^(*F*_o_^2^) + (0.0366*P*)^2^ + 0.375*P*] where *P* = (*F*_o_^2^ + 2*F*_c_^2^)/3
*S* = 1.11	(Δ/σ)_max_ = 0.006
18433 reflections	Δρ_max_ = 0.69 e Å^−^^3^
257 parameters	Δρ_min_ = −0.72 e Å^−^^3^
0 restraints	

### 6-(4-bromo-1-tosylpyrrol-2-yl)-4,4-dimethyl-5-nitrohexan-2-one (3) Special details

**Table:**

Geometry. All esds (except the esd in the dihedral angle between two l.s. planes) are estimated using the full covariance matrix. The cell esds are taken into account individually in the estimation of esds in distances, angles and torsion angles; correlations between esds in cell parameters are only used when they are defined by crystal symmetry. An approximate (isotropic) treatment of cell esds is used for estimating esds involving l.s. planes.

### 6-(4-bromo-1-tosylpyrrol-2-yl)-4,4-dimethyl-5-nitrohexan-2-one (3) Fractional atomic coordinates and isotropic or equivalent isotropic displacement parameters (Å^2^)

**Table:**

	*x*	*y*	*z*	*U*_iso_*/*U*_eq_	
Br1	0.22008 (2)	0.57357 (2)	0.01021 (2)	0.01695 (2)	
N1	0.17070 (6)	0.42535 (3)	0.20251 (3)	0.01145 (6)	
C3	0.37857 (7)	0.44721 (3)	0.11798 (3)	0.01261 (7)	
H3	0.483498	0.444262	0.091152	0.015*	
N8	0.44371 (8)	0.23904 (3)	0.11422 (3)	0.01693 (8)	
C2	0.33880 (7)	0.40180 (3)	0.18381 (3)	0.01093 (6)	
C4	0.23457 (7)	0.49959 (3)	0.09697 (3)	0.01208 (7)	
O9	0.57359 (9)	0.27017 (4)	0.08769 (4)	0.02494 (10)	
C5	0.10853 (7)	0.48676 (3)	0.14928 (3)	0.01247 (7)	
H5	−0.000347	0.514227	0.149476	0.015*	
C6	0.45096 (7)	0.33876 (3)	0.22754 (3)	0.01219 (7)	
H6A	0.434437	0.344148	0.285479	0.015*	
H6B	0.573549	0.351849	0.218744	0.015*	
C7	0.41499 (7)	0.24784 (3)	0.20244 (3)	0.01148 (7)	
H7	0.290618	0.235381	0.210853	0.014*	
O10	0.33578 (10)	0.19916 (4)	0.07358 (3)	0.02730 (12)	
C11	0.52787 (7)	0.18170 (3)	0.24936 (3)	0.01285 (7)	
C12	0.51170 (9)	0.09560 (4)	0.20840 (4)	0.01678 (9)	
H12A	0.566845	0.052934	0.243202	0.025*	
H12B	0.568869	0.097269	0.157640	0.025*	
H12C	0.389085	0.081793	0.198271	0.025*	
C13	0.71998 (8)	0.20658 (4)	0.25676 (5)	0.01915 (10)	
H13A	0.786783	0.161943	0.284110	0.029*	
H13B	0.732997	0.258568	0.287538	0.029*	
H13C	0.762620	0.215080	0.203347	0.029*	
C14	0.46432 (8)	0.17737 (4)	0.33473 (3)	0.01491 (8)	
H14A	0.542953	0.139094	0.365724	0.018*	
H14B	0.479873	0.233710	0.358656	0.018*	
C15	0.28132 (9)	0.14997 (4)	0.34817 (4)	0.01594 (8)	
O16	0.18406 (7)	0.12067 (4)	0.29612 (3)	0.02047 (8)	
C17	0.22683 (13)	0.16059 (6)	0.43234 (5)	0.02768 (15)	
H17A	0.322919	0.145112	0.469905	0.042*	
H17B	0.127148	0.124460	0.440913	0.042*	
H17C	0.194909	0.219124	0.441078	0.042*	
S18	0.05660 (2)	0.39646 (2)	0.28052 (2)	0.01139 (2)	
O19	−0.11952 (6)	0.41232 (3)	0.25512 (3)	0.01581 (7)	
O20	0.11448 (7)	0.31396 (3)	0.30178 (3)	0.01737 (7)	
C21	0.11675 (7)	0.46504 (3)	0.35854 (3)	0.01230 (7)	
C22	0.04515 (8)	0.54520 (4)	0.35859 (4)	0.01510 (8)	
H22	−0.032923	0.562991	0.316297	0.018*	
C23	0.09053 (9)	0.59848 (4)	0.42186 (4)	0.01861 (9)	
H23	0.043057	0.653310	0.422446	0.022*	
C24	0.20469 (9)	0.57294 (4)	0.48466 (4)	0.01888 (10)	
C25	0.27117 (9)	0.49166 (5)	0.48363 (4)	0.01981 (10)	
H25	0.346580	0.473194	0.526648	0.024*	
C26	0.22901 (8)	0.43731 (4)	0.42083 (4)	0.01686 (9)	
H26	0.275760	0.382327	0.420358	0.020*	
C27	0.25660 (13)	0.63236 (6)	0.55124 (5)	0.02910 (16)	
H27A	0.286189	0.600523	0.600043	0.044*	
H27B	0.160185	0.670287	0.560580	0.044*	
H27C	0.357263	0.665022	0.536490	0.044*	

### 6-(4-bromo-1-tosylpyrrol-2-yl)-4,4-dimethyl-5-nitrohexan-2-one (3) Atomic displacement parameters (Å^2^)

**Table:**

	*U*^11^	*U*^22^	*U*^33^	*U*^12^	*U*^13^	*U*^23^
Br1	0.01729 (3)	0.02109 (3)	0.01259 (2)	0.00044 (2)	0.00191 (2)	0.00605 (2)
N1	0.01097 (14)	0.01251 (15)	0.01104 (14)	0.00102 (11)	0.00231 (11)	0.00178 (11)
C3	0.01252 (17)	0.01266 (16)	0.01295 (17)	0.00042 (13)	0.00361 (13)	0.00055 (13)
N8	0.0264 (2)	0.01156 (16)	0.01297 (17)	0.00279 (15)	0.00230 (16)	0.00011 (13)
C2	0.01090 (15)	0.00970 (15)	0.01232 (16)	0.00022 (12)	0.00189 (12)	−0.00041 (12)
C4	0.01302 (17)	0.01289 (17)	0.01043 (16)	−0.00009 (13)	0.00146 (13)	0.00130 (13)
O9	0.0316 (3)	0.0223 (2)	0.0222 (2)	0.00315 (19)	0.0137 (2)	0.00241 (17)
C5	0.01182 (16)	0.01417 (17)	0.01151 (17)	0.00155 (13)	0.00149 (13)	0.00201 (13)
C6	0.01206 (16)	0.00977 (15)	0.01461 (18)	0.00011 (12)	−0.00042 (13)	−0.00072 (13)
C7	0.01306 (16)	0.00962 (15)	0.01172 (16)	0.00006 (12)	0.00023 (13)	−0.00016 (12)
O10	0.0448 (3)	0.0210 (2)	0.01518 (19)	−0.0039 (2)	−0.0072 (2)	−0.00253 (16)
C11	0.01313 (17)	0.01012 (16)	0.01516 (19)	0.00026 (13)	−0.00073 (14)	0.00104 (13)
C12	0.0209 (2)	0.01018 (17)	0.0193 (2)	0.00159 (16)	0.00143 (18)	−0.00047 (15)
C13	0.01240 (18)	0.0166 (2)	0.0282 (3)	0.00095 (16)	−0.00065 (18)	0.00158 (19)
C14	0.0173 (2)	0.01399 (18)	0.01306 (18)	−0.00250 (15)	−0.00284 (15)	0.00117 (14)
C15	0.0216 (2)	0.01344 (18)	0.01275 (18)	−0.00542 (16)	0.00065 (16)	0.00065 (14)
O16	0.0214 (2)	0.0228 (2)	0.01716 (18)	−0.01001 (16)	0.00056 (15)	−0.00288 (15)
C17	0.0392 (4)	0.0296 (3)	0.0148 (2)	−0.0130 (3)	0.0068 (2)	−0.0009 (2)
S18	0.01086 (4)	0.01178 (4)	0.01174 (5)	−0.00151 (3)	0.00243 (3)	0.00137 (3)
O19	0.01000 (13)	0.02075 (17)	0.01676 (17)	−0.00281 (12)	0.00140 (12)	0.00022 (13)
O20	0.02110 (18)	0.01187 (14)	0.01957 (18)	0.00005 (13)	0.00506 (15)	0.00389 (13)
C21	0.01169 (16)	0.01488 (18)	0.01041 (16)	−0.00063 (13)	0.00135 (13)	0.00141 (13)
C22	0.01588 (19)	0.01612 (19)	0.01330 (18)	0.00146 (15)	0.00080 (15)	−0.00038 (15)
C23	0.0214 (2)	0.0188 (2)	0.0158 (2)	−0.00135 (19)	0.00307 (18)	−0.00325 (17)
C24	0.0201 (2)	0.0256 (3)	0.01121 (19)	−0.0081 (2)	0.00354 (16)	−0.00192 (17)
C25	0.0190 (2)	0.0281 (3)	0.01208 (19)	−0.0051 (2)	−0.00187 (17)	0.00301 (18)
C26	0.0164 (2)	0.0205 (2)	0.01350 (19)	0.00019 (17)	−0.00121 (16)	0.00393 (16)
C27	0.0358 (4)	0.0369 (4)	0.0150 (2)	−0.0168 (3)	0.0046 (2)	−0.0071 (2)

### 6-(4-bromo-1-tosylpyrrol-2-yl)-4,4-dimethyl-5-nitrohexan-2-one (3) Geometric parameters (Å, º)

**Table:**

Br1—C4	1.8727 (5)	C13—H13C	0.9800
N1—C5	1.3942 (7)	C14—C15	1.5103 (9)
N1—C2	1.4054 (7)	C14—H14A	0.9900
N1—S18	1.6808 (5)	C14—H14B	0.9900
C3—C2	1.3692 (7)	C15—O16	1.2150 (8)
C3—C4	1.4226 (8)	C15—C17	1.5036 (10)
C3—H3	0.9500	C17—H17A	0.9800
N8—O9	1.2258 (9)	C17—H17B	0.9800
N8—O10	1.2275 (8)	C17—H17C	0.9800
N8—C7	1.5135 (7)	S18—O19	1.4285 (5)
C2—C6	1.4954 (7)	S18—O20	1.4307 (5)
C4—C5	1.3613 (8)	S18—C21	1.7499 (6)
C5—H5	0.9500	C21—C26	1.3944 (8)
C6—C7	1.5333 (7)	C21—C22	1.3951 (8)
C6—H6A	0.9900	C22—C23	1.3902 (9)
C6—H6B	0.9900	C22—H22	0.9500
C7—C11	1.5571 (7)	C23—C24	1.3984 (10)
C7—H7	1.0000	C23—H23	0.9500
C11—C13	1.5372 (8)	C24—C25	1.3970 (11)
C11—C12	1.5392 (8)	C24—C27	1.5033 (10)
C11—C14	1.5392 (8)	C25—C26	1.3898 (10)
C12—H12A	0.9800	C25—H25	0.9500
C12—H12B	0.9800	C26—H26	0.9500
C12—H12C	0.9800	C27—H27A	0.9800
C13—H13A	0.9800	C27—H27B	0.9800
C13—H13B	0.9800	C27—H27C	0.9800
			
C5—N1—C2	109.72 (4)	H13B—C13—H13C	109.5
C5—N1—S18	120.89 (4)	C15—C14—C11	120.01 (5)
C2—N1—S18	129.11 (4)	C15—C14—H14A	107.3
C2—C3—C4	107.72 (5)	C11—C14—H14A	107.3
C2—C3—H3	126.1	C15—C14—H14B	107.3
C4—C3—H3	126.1	C11—C14—H14B	107.3
O9—N8—O10	123.76 (6)	H14A—C14—H14B	106.9
O9—N8—C7	118.87 (6)	O16—C15—C17	121.55 (6)
O10—N8—C7	117.35 (6)	O16—C15—C14	123.66 (6)
C3—C2—N1	106.76 (4)	C17—C15—C14	114.78 (6)
C3—C2—C6	127.03 (5)	C15—C17—H17A	109.5
N1—C2—C6	126.21 (5)	C15—C17—H17B	109.5
C5—C4—C3	109.30 (5)	H17A—C17—H17B	109.5
C5—C4—Br1	125.45 (4)	C15—C17—H17C	109.5
C3—C4—Br1	125.24 (4)	H17A—C17—H17C	109.5
C4—C5—N1	106.47 (5)	H17B—C17—H17C	109.5
C4—C5—H5	126.8	O19—S18—O20	121.24 (3)
N1—C5—H5	126.8	O19—S18—N1	104.58 (3)
C2—C6—C7	114.27 (4)	O20—S18—N1	106.03 (3)
C2—C6—H6A	108.7	O19—S18—C21	108.85 (3)
C7—C6—H6A	108.7	O20—S18—C21	108.86 (3)
C2—C6—H6B	108.7	N1—S18—C21	106.24 (2)
C7—C6—H6B	108.7	C26—C21—C22	121.51 (5)
H6A—C6—H6B	107.6	C26—C21—S18	119.41 (5)
N8—C7—C6	108.79 (4)	C22—C21—S18	119.02 (4)
N8—C7—C11	108.84 (4)	C23—C22—C21	118.56 (6)
C6—C7—C11	114.54 (4)	C23—C22—H22	120.7
N8—C7—H7	108.2	C21—C22—H22	120.7
C6—C7—H7	108.2	C22—C23—C24	121.30 (6)
C11—C7—H7	108.2	C22—C23—H23	119.4
C13—C11—C12	108.80 (5)	C24—C23—H23	119.4
C13—C11—C14	106.98 (5)	C25—C24—C23	118.69 (6)
C12—C11—C14	110.62 (5)	C25—C24—C27	120.79 (7)
C13—C11—C7	112.29 (5)	C23—C24—C27	120.51 (7)
C12—C11—C7	110.53 (4)	C26—C25—C24	121.21 (6)
C14—C11—C7	107.56 (4)	C26—C25—H25	119.4
C11—C12—H12A	109.5	C24—C25—H25	119.4
C11—C12—H12B	109.5	C25—C26—C21	118.72 (6)
H12A—C12—H12B	109.5	C25—C26—H26	120.6
C11—C12—H12C	109.5	C21—C26—H26	120.6
H12A—C12—H12C	109.5	C24—C27—H27A	109.5
H12B—C12—H12C	109.5	C24—C27—H27B	109.5
C11—C13—H13A	109.5	H27A—C27—H27B	109.5
C11—C13—H13B	109.5	C24—C27—H27C	109.5
H13A—C13—H13B	109.5	H27A—C27—H27C	109.5
C11—C13—H13C	109.5	H27B—C27—H27C	109.5
H13A—C13—H13C	109.5		
			
C4—C3—C2—N1	0.72 (6)	C12—C11—C14—C15	−58.53 (7)
C4—C3—C2—C6	−179.65 (5)	C7—C11—C14—C15	62.27 (6)
C5—N1—C2—C3	−1.40 (6)	C11—C14—C15—O16	9.44 (9)
S18—N1—C2—C3	−175.24 (4)	C11—C14—C15—C17	−171.28 (6)
C5—N1—C2—C6	178.97 (5)	C5—N1—S18—O19	27.62 (5)
S18—N1—C2—C6	5.13 (8)	C2—N1—S18—O19	−159.14 (5)
C2—C3—C4—C5	0.21 (7)	C5—N1—S18—O20	156.84 (5)
C2—C3—C4—Br1	−179.12 (4)	C2—N1—S18—O20	−29.92 (6)
C3—C4—C5—N1	−1.05 (6)	C5—N1—S18—C21	−87.44 (5)
Br1—C4—C5—N1	178.27 (4)	C2—N1—S18—C21	85.80 (5)
C2—N1—C5—C4	1.52 (6)	O19—S18—C21—C26	144.02 (5)
S18—N1—C5—C4	175.95 (4)	O20—S18—C21—C26	9.94 (6)
C3—C2—C6—C7	−96.18 (6)	N1—S18—C21—C26	−103.86 (5)
N1—C2—C6—C7	83.39 (7)	O19—S18—C21—C22	−33.26 (5)
O9—N8—C7—C6	45.57 (7)	O20—S18—C21—C22	−167.34 (5)
O10—N8—C7—C6	−135.92 (6)	N1—S18—C21—C22	78.86 (5)
O9—N8—C7—C11	−79.85 (6)	C26—C21—C22—C23	1.22 (9)
O10—N8—C7—C11	98.66 (6)	S18—C21—C22—C23	178.44 (5)
C2—C6—C7—N8	59.57 (6)	C21—C22—C23—C24	−0.29 (10)
C2—C6—C7—C11	−178.41 (5)	C22—C23—C24—C25	−1.06 (10)
N8—C7—C11—C13	76.03 (6)	C22—C23—C24—C27	178.05 (6)
C6—C7—C11—C13	−45.96 (7)	C23—C24—C25—C26	1.54 (10)
N8—C7—C11—C12	−45.66 (6)	C27—C24—C25—C26	−177.57 (7)
C6—C7—C11—C12	−167.66 (5)	C24—C25—C26—C21	−0.65 (10)
N8—C7—C11—C14	−166.52 (4)	C22—C21—C26—C25	−0.76 (9)
C6—C7—C11—C14	71.48 (6)	S18—C21—C26—C25	−177.96 (5)
C13—C11—C14—C15	−176.89 (5)		
